# The crystal structure of methanogen McrD, a methyl‐coenzyme M reductase‐associated protein

**DOI:** 10.1002/2211-5463.13848

**Published:** 2024-06-14

**Authors:** Andrew J. Sutherland‐Smith, Vincenzo Carbone, Linley R. Schofield, Bryan Cronin, Evert C. Duin, Ron S. Ronimus

**Affiliations:** ^1^ School of Natural Sciences Massey University Palmerston North New Zealand; ^2^ AgResearch Ltd. Grasslands Palmerston North New Zealand; ^3^ Department of Chemistry and Biochemistry Auburn University AL USA

**Keywords:** ferredoxin‐like, McrD, methanogen, *Methanomassiliicoccus luminyensis*, methyl‐coenzyme M reductase

## Abstract

Methyl‐coenzyme M reductase (MCR) is a multi‐subunit (α_2_β_2_γ_2_) enzyme responsible for methane formation via its unique F_430_ cofactor. The genes responsible for producing MCR (*mcrA*, *mcrB* and *mcrG*) are typically colocated with two other highly conserved genes *mcrC* and *mcrD*. We present here the high‐resolution crystal structure for McrD from a human gut methanogen *Methanomassiliicoccus luminyensis* strain B10. The structure reveals that McrD comprises a ferredoxin‐like domain assembled into an α + β barrel‐like dimer with conformational flexibility exhibited by a functional loop. The description of the *M. luminyensis* McrD crystal structure contributes to our understanding of this key conserved methanogen protein typically responsible for promoting MCR activity and the production of methane, a greenhouse gas.

## Graphical abstract

Methyl‐coenzyme M reductase (MCR) is responsible for biological methanogenesis. McrD and MCR are conserved and colocated in methanogen genomes, with McrD protein able to form a complex with MCR. We present the high‐resolution McrD crystal structure of ferredoxin‐like domain monomers assembled into an α + β barrel‐like dimer, with conformational heterogeneity exhibited by a key loop. Created with BioRender.com.

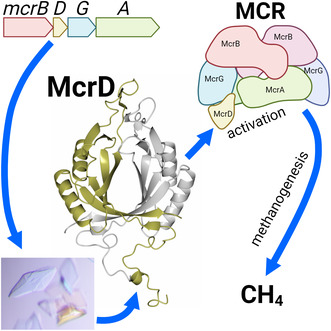

**Abbreviations**
ANKA: anaerobic alkane oxidising
ANME: anaerobic methanotrophic
AOM: anaerobic oxidation of methane
cryo‐EM: cryogenic electron microscopy
*Ma*McrD: *Methanosarcina acetivorens* McrD
MCR: Methyl‐coenzyme M reductase
*Ml*McrD: *Methanomassiliicoccus luminyensis McrD*
RMSD: root mean square difference

All biologically produced methane (methanogenesis) is produced by the archaeal enzyme methyl‐coenzyme M reductase (MCR) [1, 2], while archaea taxa that undergo anaerobic methane oxidation (AOM) use MCR in the reverse direction to methanogenesis to catalyse the initial activation of methane [3, 4]. MCR is a multi‐subunit heterohexameric enzyme (α_2_β_2_γ_2_) forming two identical active sites each with a unique nickel‐containing tetrapyrrole F_430_ cofactor bound [3, 5, 6]. The post‐translational assembly and activation of multimeric MCR into its functional enzyme form involves four processes: F_430_ biosynthesis [7, 8], enzyme‐catalysed reduction of the F_430_ nickel ion by the activation pathway [9], post‐translational modification of MCR [6, 10, 11, 12] and assembly of hexameric (α_2_β_2_γ_2_) MCR [13, 14, 15] including F_430_ insertion.

For methanogen and anaerobic methanotrophic ANME‐2 genomes, the MCR genes (*mcrBGA*) are almost always colocated with two other genes, *mcrC* and *mcrD* (*mcrBDCGA*) [16, 17, 18]. The *mcrC* gene is located at a different locus in ANME‐1 genomes while *mcrD* is not present in ANME‐1 and anaerobic alkane oxidising archaeal ANKA genomes [19]. McrC has been identified as a component of the A3a multienzyme complex in the F_430_ nickel reduction pathway for MCR activation [9]. McrD has been shown to interact with MCR in immunoprecipitation and co‐expression experiments leading to the proposal that McrD acts as a chaperone in MCR/F_430_ assembly, facilitating MCR post‐translational modification and F_430_ insertion [14, 15, 20, 21]. McrD was able to alleviate product inhibition in the final step in F_430_ biosynthesis catalysed by enzyme CfbE [7]. McrD is not present with MCR when MCR is purified on the basis of activity, it is not required for MCR *in vitro* methane production and its deletion in the *Methanosarcina acetivorens* genome had only a minor effect on cell doubling time [13, 15, 22, 23]. Affinity purification and cryo‐electron microscopy analysis has shown that *M. acetivorens* McrD (*Ma*McrD) binds MCR during actively dividing cell growth acting to produce a MCR assembly intermediate structure. The cryo‐electron microscopy image reconstruction of the McrD‐MCR complex showed a single McrD molecule bound to the MCR α_2_β_2_γ_2_ hexamer [15].

In order to obtain a greater understanding of McrD biology, we have determined the high‐resolution crystal structure of McrD from the human gut methanogen *Methanomassiliicoccus luminyensis*. The McrD structure contains a ferredoxin‐like fold assembled in a dimeric α + β barrel structure, with a key functional loop that can adopt alternate conformations. The McrD crystal structure adds insight into its role as a chaperone in the assembly of the MCR complex and its cofactors enhancing our understanding of the processes that ultimately lead to methane formation.

## Materials and methods

### Cloning, expression and purification of McrD

The *mcrD* gene from *M. luminyensis* strain B10 (NCBI accession: WP_019176772.1) was cloned into the TOPO pET151D expression plasmid, essentially as described previously [24]. Briefly, forward 5′‐CACCATGAGTACTGACAAATTTGAACCG and reverse primers 5′‐TTACCTCTTGCCTTTCTTATAATCG were used in a PCR reaction to amplify the *mcrD* cDNA which was then inserted into pET151D (LifeTechnologies, Carlsbad, CA, USA) using topoisomerase‐mediated cloning. Colony PCR was used to identify positives clones, and the resulting plasmid purified and sequenced to verify successful cloning. *Escherichia coli* BL21 (strain LOBSTR; Kerafast, Boston, MA, USA) were transformed with the pET151D‐McrD plasmid and grown with 1 mm IPTG added to induce McrD expression. Nickel nitriloacetic acid affinity chromatography was performed on the cell lysate containing hexa‐histidine tagged McrD from the transformed and cultured *E. coli*. The imidazole‐eluted McrD‐containing fractions were dialysed into a final storage buffer (20 mm MOPS buffer, pH 7, 50 mm NaCl, 2 mm β‐mercaptoethanol, 2 mm TCEP) and concentrated to 22 mg·mL^−1^. SDS‐gel protein electrophoresis confirmed a high level of purity for the protein preparation.

### McrD crystal structure determination

Plate‐like McrD crystals were obtained in 0.03 m MgCl_2_, 0.03 m CaCl_2_, 0.05 m MES, 0.05 m imidazole, pH 6.5, 20% glycerol, 10% PEG 4000, 0.02 m carboxylic acids mix (formate, acetate, citrate, tartrate and oxamate; solution G3 from the Morpheus I Crystal Screen (Molecular Dimensions, Rotherham, UK)) at 4 °C after 3–4 days. Crystals were flash frozen in the presence of additional glycerol to a final concentration of 25% (v/v) or ethylene glycol as cryoprotectant and diffraction data collected at the Australian Synchrotron MX2 beamline using an Eiger detection system [25]. X‐ray diffraction data were integrated with xds [26] before being scaled and averaged with pointless/aimless [27, 28]. The McrD structure was determined by single isomorphous replacement with anomalous scattering (SIRAS) using shelx [29] as implemented in crank2 [30]. SIRAS derivative data were collected on a crystal soaked for 20 s in mother liquor containing 25% (v/v) ethylene glycol and 0.5 m NaI. The resulting interpretable electron density map was autobuilt with buccaneer [31], followed by cycles of manual building and refinement with coot [32] and refmac5 [33] respectively, with a final *R* and *R*_free_ of 0.178 and 0.192 at 1.65 Å resolution. X‐ray diffraction and refined structure statistics are provided in Table 1. Figures were prepared with ccp4mg [34] and pymol [35]. Protein oligomerisation analysis was performed with pisa [36].

**Table 1:** Crystallographic X‐ray diffraction data and refined structure statistics.

	Native			NaI derivative		
	Overall	Inner	Outer	Overall	Inner	Outer
Low‐resolution limit (Å)	45.11	45.11	1.68	44.93	44.93	2.32
High‐resolution limit (Å)	1.65	9.04	1.65	2.24	8.97	2.24
*R*_merge_ (all *I*+ and *I*−)	0.072	0.048	2.333	0.086	0.034	1.098
*R*_meas_ (all *I*+ and *I*−)	0.075	0.050	2.420	0.089	0.036	1.141
*R*_pim_ (all *I*+ and *I*−)	0.020	0.015	0.641	0.024	0.01	0.307
*R*_merge_ in top intensity bin	0.039			0.035		
Number of observations	784 283	4905	39 087	313 820	5029	27 492
Number unique reflections	57 078	402	2792	23 052	411	2042
Mean((*I*)/SD(*I*))	16.8	51.7	1.6	14.3	49.6	1.9
Half‐set correlation CC_(1/2)_	1.00	1.00	0.82	1.00	1.00	0.91
Completeness (%)	100.0	99.4	100.0	99.7	99.3	96.8
Multiplicity	13.7	12.2	14.0	13.6	12.2	13.5
Anomalous completeness (%)	100.0	99.6	100.0	99.6	99.6	95.6
Anomalous multiplicity	7.1	7.4	7.2	7.1	7.4	7.0
*R*‐factor	0.178					
*R*‐free	0.192					
RMSD bonds (Å)	0.007					
RMSD angles (°)	1.51					
Ramachandran favoured residues	98.3%					
Ramachandran allowed residues	1.7%					

## Results

**Fig. 1:**
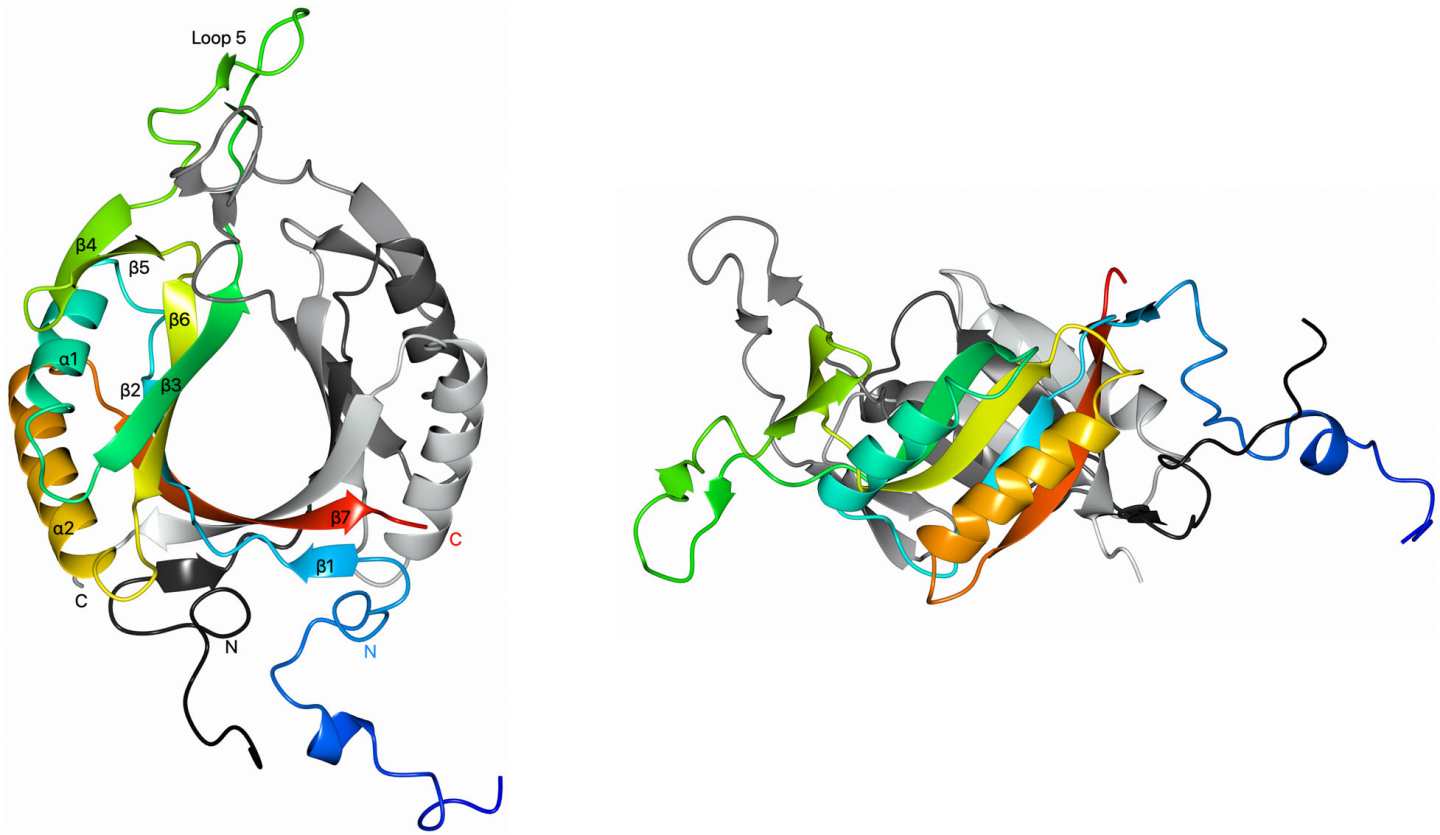
Orthogonal views of the *M. luminyensis* McrD dimer crystal structure in cartoon representation. Monomer A is sequence colour‐ramped from the N terminus (dark blue) to the C terminus (red) with secondary structure elements labelled. Monomer B is grey scale‐ramped from the N terminus (black) to the C terminus (light grey). N and C label the McrD N terminus (amino acid 1) and C terminus (amino acid 129/130), respectively.

The high‐resolution (1.65 Å) McrD crystal structure has a ferredoxin‐like fold with a signature core βαβ‐βαβ topology fold corresponding to the β2α1β3‐β6α2β7 secondary structure elements (Fig. 1). There is an extended glycine‐rich loop region (loop 5, amino acids 49–69; 6 glycines) and a 2‐stranded β‐sheet (β‐strands 4 and 5) between the two βαβ motifs. A short β‐strand (β1) at the N terminus forms an anti‐parallel interaction with the C‐terminal β‐strand (β7). There are 18 and 9 amino acids present at the N terminus of monomer A and B, respectively, from the pET151D affinity tag that have interpretable electron density and are included in the structure.

**Fig. 2:**
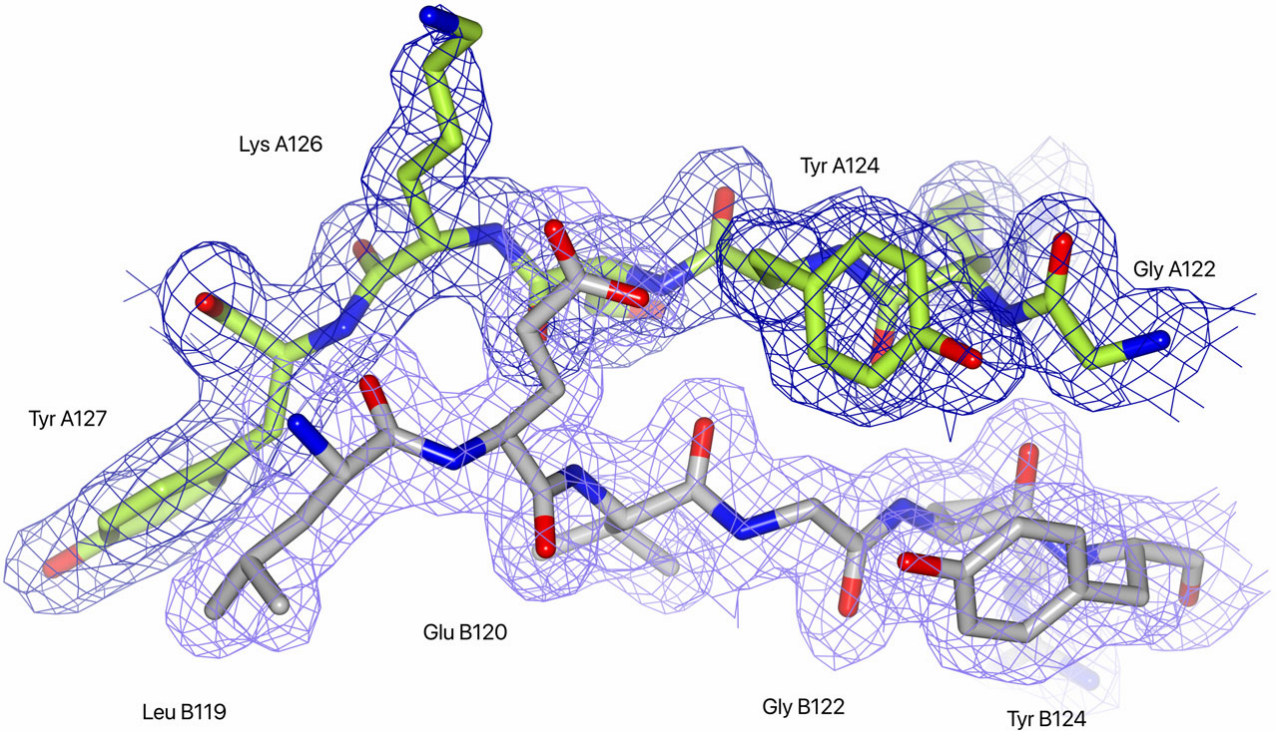
The McrD electron density across a region of the dimer interface. The McrD structure is shown in stick representation coloured by atom type; carbon atoms are coloured green or grey for monomer A and monomer B respectively, oxygen are red and nitrogen are blue. The weighted *2fo‐fc* electron density is contoured at 1 sigma and clipped to the atoms shown at a radius of 1.5 Å.

**Fig. 3:**
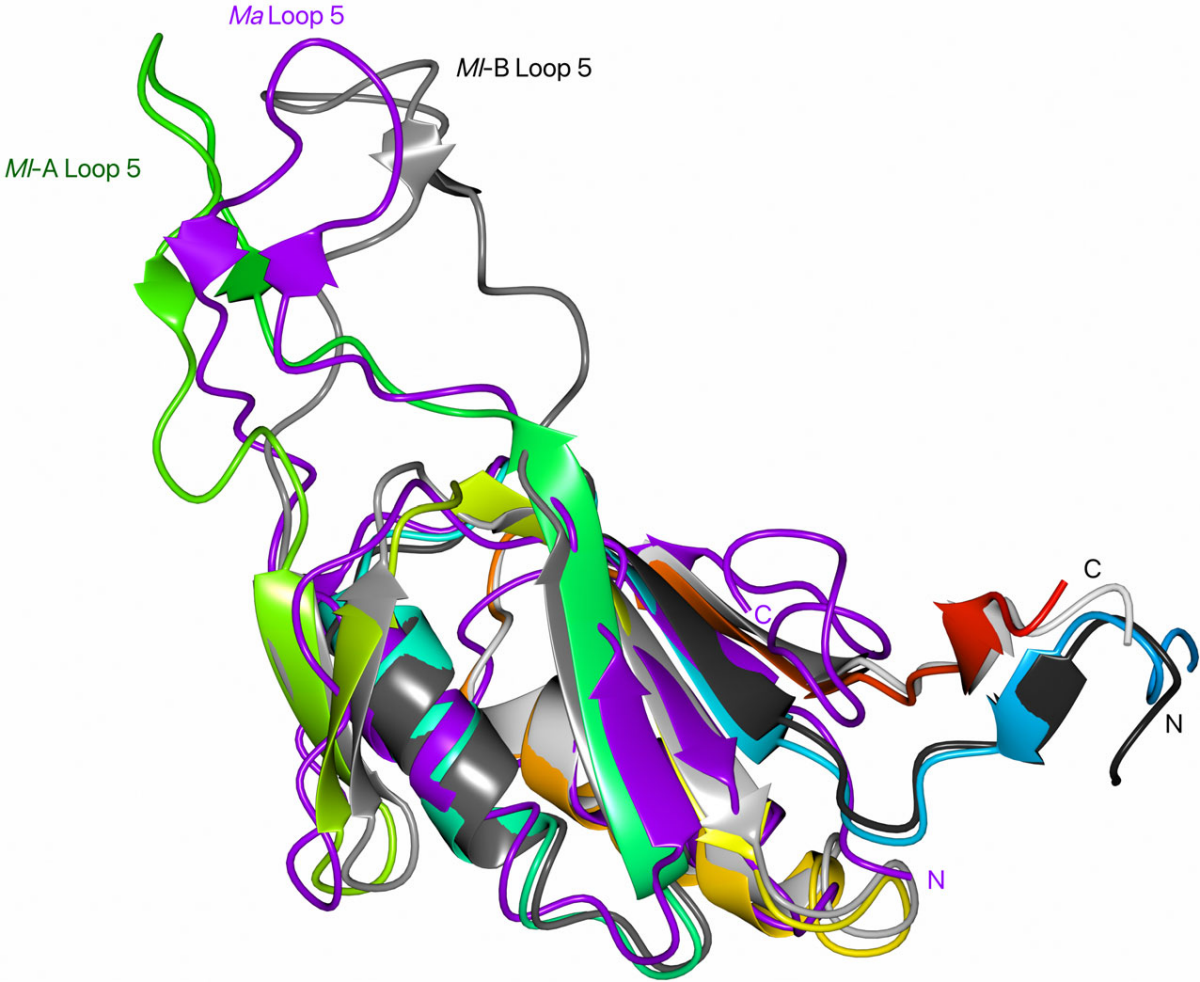
Superposition of the *M. luminyensis* (*Ml*) and *M. acetivorans* (*Ma*) McrD structures in cartoon representation. *Ml*McrD monomer A (*Ml*‐A) is colour‐ramped N to C terminus from blue to red, *Ml*McrD monomer B (*Ml*‐B) is grey scale‐ramped N to C termini black to light grey *Ma*McrD (*Ma*) (PDB: 8GF6) [15] is shown in purple. Loop 5 and the termini are labelled. The *Ml*McrD amino acids within the plasmid‐encoded affinity tag were not included in the superposition and are not shown.

McrD crystallised in C222_1_ space group with a unit cell containing two molecules in the asymmetric unit that form a dimer with an interface area of 1822 Å^2^. There are no surfaces indicative of any higher order oligomerisation. The McrD dimer forms a three‐quarter β‐barrel structure with each monomer contributing an anti‐parallel curved four‐stranded β‐sheet from the core ferredoxin‐like domain fold (β‐strands 2, 3, 6, 7) to the β‐barrel with the α‐helices external. The C‐terminal (fourth) core ferredoxin‐like domain β‐strand (β7, amino acids 119–127) forms an anti‐parallel interaction across the dimer interface with the non‐crystallographic symmetry related C‐terminal β7 strand of the other monomer (Figs 1 and 2). There are additional dimer contacts between the extended loop 5 region and between α2 and the N terminus. The McrD dimeric crystal structure aligns with the dimeric α + β barrel superfamily (PFAM CL0032 SCOPe (v2.08) superfamily d.58.454909) [37]. Two types of packing for dimeric or fused ferredoxin α + β barrels have been described [38]; the McrD dimer has elements of type 2 packing with the C‐terminal β‐strands (β7) of each monomer forming a large proportion of the dimer interface, but the three‐quarter nature of the McrD barrel means that the β3 strands do not associate to complete the barrel structure, instead this position in the McrD dimer is occupied by loop 5 from each monomer (Fig. 1). The two monomers of the McrD dimer superimpose with a high degree of structural similarity within their core regions excluding loop 5 which adopts a different conformation in each monomer (Fig. 3), largely as a result of a rigid body shift (root mean square difference RMSD of 0.9 Å for Cα 1–48 and 70–129). The McrD monomers superimpose on one another with RMSD 3.0 Å (117 Cα) when loop 5 is included.

**Fig. 4:**
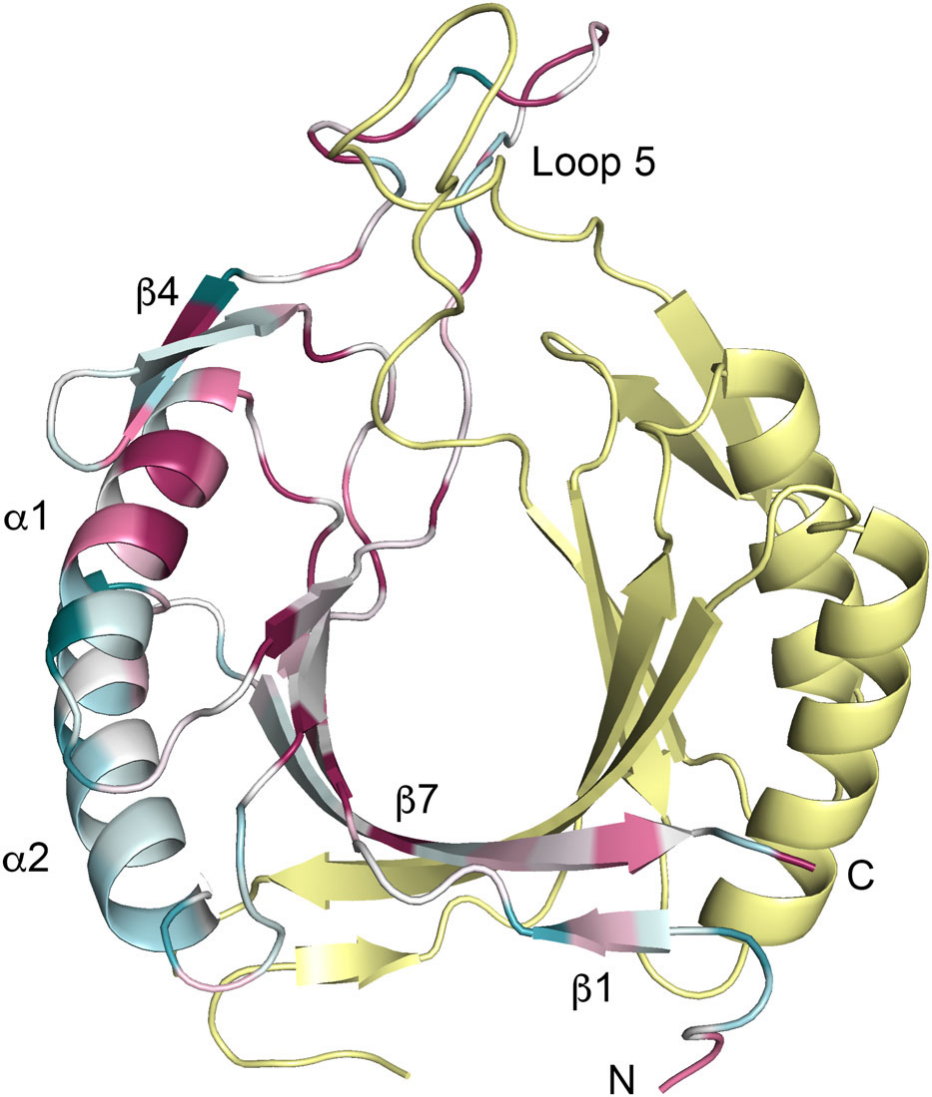
McrD ConSurf [40] sequence conservation analysis mapped on to the *M. luminyensis McrD* crystal structure. The dimer structure is shown in cartoon representation with monomer A colour‐ramped by sequence conservation as calculated from 150 sequences (dark magenta most conserved, white intermediate and cyan least conserved). Monomer B is coloured yellow. The N‐terminal plasmid tag‐encoded amino acids in the structure were removed for this analysis.

NCBI Blast multiple sequence alignment [39] and ConSurf [40] analyses of homologous McrD sequences mapped to the *M. luminyensis* McrD structure did not reveal any obvious conserved surfaces that might be indicative of functional regions (Fig. 4). A section of helix α1 (amino acids 26–31) and residues within or nearby loop 5 are conserved (G47, P51, G57, P58, G61, V64 and H66) suggesting that the properties of these elements are important for McrD structure and/or function. There are also a number of conserved residues at the dimer interface (R69, L81, G122, R123, Y124, K126 and P129 C terminus) and other conserved residues dispersed across the structure as a mixture of surface exposed (R20, Q42, K70, G86) and buried residues (E13, L22, I72 and E91). Helix α2 is the most sequence variable region. The positions of the *Ml*McrD C‐terminal residues (10 for monomer A and 9 for monomer B) are not interpretable in the electron density maps and consequently are not modelled. The *Ml*McrD sequence is 139 amino acids in length; residues C‐terminal to the highly conserved P129 (Fig. 4) are presumed to extend in a disordered manner away from the core α + β barrel structure into a solvent channel within the crystal lattice.

## Discussion

The crystal structure of McrD shows that it has a core βαββαβ ferredoxin‐like fold with an extended loop 5 region and two β‐strands inserted between the two βαβ motifs. The MCR α and β subunit N‐terminal domains, and the γ subunit, also contain a ferredoxin‐like fold that are decorated with additional elements [5, 41] placing McrA (Mcr α subunit), McrB (β subunit), McrG (γ subunit) and McrD all in the same SCOPe Fold classification [37]. The ferredoxin‐like fold is considered to be an ancient domain central to the origin of metabolic pathways, including methanogenesis [42, 43]. The ferredoxin‐like domain is a common fold in archaea and is also widespread in eukaryotes and bacteria [44]. Structural homology analysis using dali [45], salami [46] and foldseek [47] identified numerous structurally related ferredoxin‐like domain proteins as homologues of McrD, including some dimeric proteins, but these analysis methods did not identify any consensus homologues. This result likely reflects the widespread presence of ferredoxin‐like domains within proteomes, the non‐typical α + β barrel dimer McrD structure and also the specialised functional role of McrD. Comparison of the two molecules in the *Ml*McrD dimeric crystal structure reveals that loop 5 adopts a different conformation relative to the core ferredoxin‐like domain for each of the two monomers.

The *M. acetivorans* McrD‐MCR complex structure determined by cryo‐electron microscopy shows that McrD interacts asymmetrically as a single molecule with the MCR α_2_β_2_γ_2_ hexamer [15]. *Ma*McrD binds to MCR in both the apo‐apo (free of F_430_, CoM and CoB cofactors) or semi‐apo where the cofactors are present in a single active site suggesting that McrD assists in the assembly of an intermediate MCR state [15]. *Ma*McrD binds the MCR α_2_β_2_γ_2_ hexamer at the interface of the α, α′ (α′ is the second α subunit of the MCR α_2_β_2_γ_2_ hexamer) and γ subunits with the McrD loop 5 located between the MCR α′ and γ subunits, occupying the position normally filled by the MCR α subunit N‐terminal domain when McrD is not bound. The displaced MCR α subunit N‐terminal domain forms a β‐strand that inserts into the McrD β‐sheet in a parallel manner alongside strand β4 [15]. Superposition of the *Ma*McrD structure fitted to the *Ma*McrD/MCR cryo‐EM image reconstruction to that of the *Ml*McrD monomers reveals that the core ferredoxin‐like fold is conserved but reveals a conformational difference in the large loop 5 (Fig. 3). The *Ma*McrD structure superimposes with *Ml*McrD monomers A and B with RSMD values of 2.7 Å (102 Cα) and 4.9 Å (109 Cα), respectively. The structural diversity of loop 5 shown by *Ml*McrD monomers A and B, and *Ma*McrD at the interaction interface with MCR, suggests this structural heterogeneity is likely to be important in its MCR‐binding role [15].

The significance of the dimer in the function of *Ml*McrD is unclear at this stage given that *Ma*McrD binds MCR as a single molecule. Superposition homology modelling analysis shows that dimeric *Ml*McrD would not able to bind the MCR complex as a dimer in an homologous way to *Ma*McrD owing to steric clashes from the second *Ml*McrD monomer, and even though the MCR α, β and γ subunits all contain ferredoxin‐like domains within their structures the *Ml*McrD dimer interface does not match the interaction interface between monomeric *Ma*McrD and MCR. *Ml*McrD has a shorter sequence than *Ma*McrD, and most other McrD proteins, with a C‐terminal deletion of an approximately 30 amino acid domain. The functional significance of this McrD C‐terminal domain is unclear considering its lack of absolute conservation and it not being observed in the *Ma*McrD/MCR complex cryo‐electron microscopy image reconstruction [15].

Overall, the *M. luminyensis* McrD crystal structure adds additional structural insights into the biology of this key conserved methanogen protein responsible for promoting MCR activity and methane greenhouse gas production.

## Conflict of interest

The authors declare no conflict of interest.

### Peer review

The peer review history for this article is available at https://www.webofscience.com/api/gateway/wos/peer-review/10.1002/2211-5463.13848.

## Author contributions

AJS‐S, VC, LRS, BC, ECD and RSR were responsible for the experimental design, and conducted the experimental analysis. AJS‐S, VC, LRS and RSR drafted the manuscript. AJS‐S, VC, LRS, ECD and RSR revised the manuscript.

## Data Availability

The structural data that support these findings are openly available in the wwPDB at https://doi.org/10.2210/pdb8W33/pdb.
